# Safety and Efficacy of Laser Interstitial Thermal Therapy as Upfront Therapy in Primary Glioblastoma and IDH-Mutant Astrocytoma: A Meta-Analysis

**DOI:** 10.3390/cancers16112131

**Published:** 2024-06-03

**Authors:** Aryan Pandey, Anubhav Chandla, Mahlet Mekonnen, Gabrielle E. A. Hovis, Zoe E. Teton, Kunal S. Patel, Richard G. Everson, Madhuri Wadehra, Isaac Yang

**Affiliations:** 1 Department of Neurosurgery, University of California Los Angeles (UCLA), Los Angeles, CA 90095, USAmahlet44@gmail.com (M.M.);; 2 Harbor-UCLA Medical Center, Torrance, CA 90502, USA; 3 Jonsson Comprehensive Cancer Center, Los Angeles, CA 90095, USA; 4 Department of Radiation Oncology, University of California Los Angeles (UCLA), Los Angeles, CA 90095, USA; 5 The Lundquist Institute for Biomedical Innovation at Harbor-UCLA Medical Center, Torrance, CA 90502, USA; 6 Ronald Reagan UCLA Medical Center, Los Angeles, CA 90095, USA; 7 Department of Pathology and Laboratory Medicine, University of California Los Angeles (UCLA), Los Angeles, CA 90095, USA; 8 Department of Head and Neck Surgery, University of California Los Angeles (UCLA), Los Angeles, CA 90095, USA

**Keywords:** primary brain tumors, glioblastoma, malignant gliomas, 5-ALA, LITT, meningioma, skull-based tumors, molecular targeted therapy, immunotherapy, vaccine therapy

## Abstract

**Simple Summary:**

Laser interstitial thermal therapy (LITT) is a minimally invasive, MRI-guided procedure that causes localized tissue hyperthermia. This results in the ablation of targeted cells and disruption of the blood–brain barrier. LITT offers an alternative treatment option to standard-of-care resection for glioma in addition to potentially enhancing drug delivery of adjuvant therapy. Here, we present the first meta-analysis since the fifth edition of the WHO Classification of Tumors of the Central Nervous System (WHO CNS5) to assess the current literature on the safety and efficacy of LITT in the upfront treatment of primary brain tumors. This study summarizes the current relevant literature on LITT and supports its viability as a treatment option for glioblastoma and IDH-mutant astrocytoma.

**Abstract:**

Although primary studies have reported the safety and efficacy of LITT as a primary treatment in glioma, they are limited by sample sizes and institutional variation in stereotactic parameters such as temperature and laser power. The current literature has yet to provide pooled statistics on outcomes solely for primary brain tumors according to the 2021 WHO Classification of Tumors of the Central Nervous System (WHO CNS5). In the present study, we identify recent articles on primary CNS neoplasms treated with LITT without prior intervention, focusing on relationships with molecular profile, PFS, and OS. This meta-analysis includes the extraction of data from primary sources across four databases using the Covidence systematic review manager. The pooled data suggest LITT may be a safe primary management option with tumor ablation rates of 94.8% and 84.6% in IDH-wildtype glioblastoma multiforme (GBM) and IDH-mutant astrocytoma, respectively. For IDH-wildtype GBM, the pooled PFS and OS were 5.0 and 9.0 months, respectively. Similar to rates reported in the prior literature, the neurologic and non-neurologic complication rates for IDH-wildtype GBM were 10.3% and 4.8%, respectively. The neurologic and non-neurologic complication rates were somewhat higher in the IDH-mutant astrocytoma cohort at 33% and 8.3%, likely due to a smaller cohort size.

## 1. Introduction

In 2021, the World Health Organization (WHO) reclassified primary central nervous system (CNS) tumors by incorporating molecular markers, as opposed to solely relying on histologic and genetic features as they had in years past [1,2,3,4]. This integration of novel diagnostic technologies, including next-generation sequencing and DNA methylation profiling, resulted in the publication of the fifth edition of the WHO Classification of Tumors of the CNS (WHO CNS5) [3,5]. The updated nomenclature recategorizes IDH-mutant glioblastoma (GBM) without a 1p/19q co-deletion under the umbrella of IDH-mutant astrocytoma that may be classified as WHO grade 2–4 depending on further molecular profiling. Instead, GBM became defined by its IDH-wildtype status, as it is commonly recognized today [3,6,7]. While advancements in neuroimaging techniques and the implementation of 5-ALA in intraoperative fluorescence mapping have augmented surgeons’ ability to obtain gross total resection, instances remain in which surgery is not an option or not preferred by the patient [7,8,9,10,11,12]. Furthermore, certain factors are associated with reduced response to resection with adjuvant chemoradiation, such as IDH-wildtype GBM without methylguanine methyltransferase (MGMT) promoter methylation, poor tumor excisability, and resistance to chemotherapy [13,14]. In cases of unresectable tumors, resistance to adjuvant therapies, deep lesions, significant comorbidity, and, to an extent, tumors located proximal to eloquent structures, minimally invasive laser-interstitial thermal therapy (LITT) is considered an effective alternative treatment modality for primary CNS tumors [15,16].

LITT (also known as stereotactic laser ablation) involves stereotactic navigation in delivering laser energy using an inserted optical fiber that ablates the tumor and has secondary effects of forming a hypoxic antitumor microenvironment by local hyperthermia, cellular necrosis, and focal coagulation [17,18,19]. The earliest case of LITT for brain tumors was reported in 1983, but its efficacy was limited [15,20,21]. The later development of magnetic resonance-guided LITT (MR-LITT) introduced high-resolution imaging with real-time magnetic resonance imaging (MRI) thermometry and spurred the widespread application of LITT for the management of CNS tumors, radiation necrosis, and epilepsy [15,22]. Given its minimally invasive nature, LITT is often preferable in patients with significant comorbidities, deep-seated lesions, low functional scores, or, to an extent, an inability to tolerate anesthesia [23,24]. Relative to standard open surgery for tumor resection, LITT is hypothesized to have equivalent efficacy with improved recovery time, shorter hospital stay, and lower physiologic impact of blood loss [12,25,26]. Although LITT may be complicated by hemorrhage, seizure, edema, and radiation to neighboring tissue, the incidence rate of these complications is generally lower than in other invasive treatment modalities [12].

Currently, there are multiple studies investigating the possibility of multimodal treatment in combination with LITT to provide synergistic delivery of other therapeutics to the tumor microenvironment [27,28,29,30,31]. Beyond just directed tumor cell death, LITT is also hypothesized to decrease the stability of tight junctions and the rate of transcytosis across the blood–blood–brain barrier (BBB) [32,33]. In doing so, LITT increases the permeability of the BBB and enables the greater delivery of therapeutics to target CNS neoplasms by presumptively increasing chemokine production and antigen presentation [32,34,35]. In this way, LITT offers a unique advantage in comparison to non-stereotactic treatment modalities by enhancing the delivery of immunotherapies, as well as other treatments [36,37,38,39,40,41,42,43].

Over the years, several retrospective and prospective studies have reported the efficacy of LITT [44,45,46]. Here, we present a comprehensive summary of the most recent advances since WHO CNS5 was released in LITT as an upfront treatment for primary tumors.

## 2. Materials and Methods

### 2.1. Data Collection

Original studies using LITT for primary brain neoplasms were identified per the Preferred Reporting Items for Systematic Reviews and Meta-Analyses (PRISMA) guidelines [47]. Boolean combinations of (Primary Brain Tumor OR Central Nervous System Tumor OR Brain Tumor OR Intracranial Tumor OR Intracerebral Neoplasm) AND (LITT OR Laser Interstitial Thermal Therapy) were queried across four databases (PubMed, Embase, Cochrane, and Web of Science). All studies were included based on LITT’s first descriptions from 1983 and after with no specified time range [48]. Two independent reviewers screened all studies using the Covidence systematic review manager. Articles reporting primary tumors treated with LITT without prior intervention in the adult patient population were included. The term ‘primary’ included all non-recurrent, non-metastatic tumors that originated in the brain. Non-human, pediatric, and pregnant patients were excluded, as were duplicated articles, non-English manuscripts, case reports, and review articles.

### 2.2. Data Extraction

All reported data from studies was extracted using the Covidence systematic review manager. Demographic data such as age, sex, tumor type, and other pertinent clinical data were included. LITT-specific variables of interest included laser type, laser developer, equipment power, and other equipment specifications. Preoperative variables of interest included preoperative tumor volume, tumor location, tumor laterality, methylation status, mutational profile, and preoperative Karnofsky performance score. Clinical outcomes included postoperative tumor volume, the extent of ablation, and resulting complications. To consolidate heterogeneous descriptions of postoperative complications, adverse events pertaining to neurologic outcomes were defined as “neurologic complications”, which comprised “transient aphasia”, “seizure”, “motor function deficits”, and “hemiparesis”. Some studies grouped their neurologic complications and reported them only as ‘neurologic deficit’ without further specification. These have been grouped together as “non-specified neurologic deficits”. Other complications were deemed “non-neurologic complications” which comprised: “edema”, “hemorrhage”, “hydrocephalus”, “pulmonary embolism”, “DVT”, “wound infection”, and “meningitis”. Overall survival, progression-free survival, and mortality rates were also recorded.

### 2.3. Statistical Analysis

**Figure 1:**
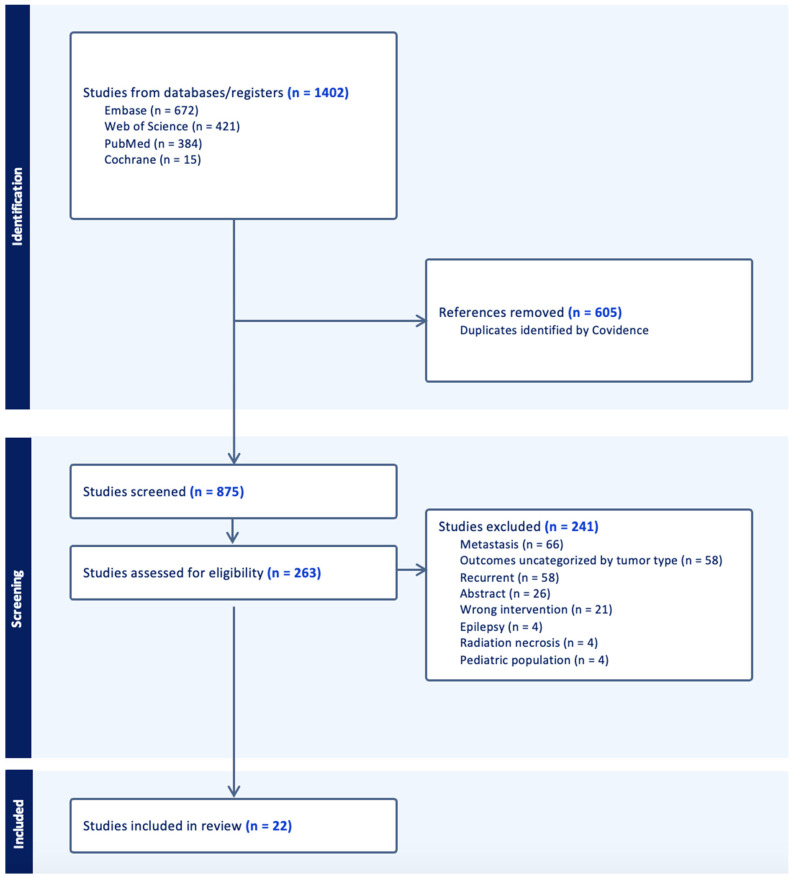
PRISMA flowchart for the filtering of 1402 primary scientific articles into 22 studies included in the meta-analysis.

All data were analyzed using the “meta” function in Stata 18 Standard Edition (SE) [49,50,51]. During the abstract screening, single patient reports and studies were not included in the analysis due to limited sample size (Figure 1). Due to the limited sample size of studies reporting primary meningiomas and oligodendrogliomas, we excluded them. Overall, we statistically analyzed two cohorts: patients diagnosed with IDH-wildtype GBM, and patients diagnosed with IDH-mutant astrocytoma. Descriptive data were reported as means with standard deviations. Due to limited data availability across selected studies, pooled proportional analysis was performed to calculate pooled percentages for all binomial study characteristics. Study heterogeneity was quantified via the Cochrane Q statistic. Forest plots were used to summarize individual study proportions, study heterogeneity, and the pooled proportional effects observed throughout the study. Meta-regressions were used to evaluate the correlations between proportional data and continuous variables of interest while subgroup analysis was performed amongst categorical data evaluating proportional data of interest. All binary data were reported as pooled percentages while continuous data were reported as pooled means with standard deviations. Publication bias was evaluated via contour-enhanced funnel plots at contour significance levels of 1%, 5%, and 10%. Egger’s test was utilized to evaluate if funnel plot asymmetry leading to publication bias was statistically significant. Random effects modeling (REML) was utilized to account for inter-study heterogeneity. Sensitivity analysis of outcomes with no significant study heterogeneity was performed if the Cochrane Q statistic was insignificant (*p* > 0.05) using a common-effects inverse variance model. A *p*-value ≤ 0.05 was deemed significant for all other statistical testing.

## 3. Results

### 3.1. Search Results

Following the specified search criteria, a total of 875 deduplicated studies were screened for inclusion (Figure 1). Following the abstract screening, 621 studies were excluded if the inclusion criteria was not met, and the search terms were not appropriately included. During full text screening, the top reasons why we excluded studies involved the reporting of pooled data in which outcomes for metastatic tumors were not differentiated, were not stratified by tumor type, or stratified for recurrent and primary cohorts. Ultimately, we excluded another 241 studies and 22 were included after the completion of the full-text review. A majority of the studies were screened out due to presenting data that were unstratified by tumor type, metastatic tumors, and recurrent tumors. Among this cohort of 206 patients, 16 studies included LITT for primary IDH-wild type glioblastoma (N = 185, 90%), while 6 studies reported on LITT for IDH-mutant astrocytoma (N = 21, 10%) (Table 1).

### 3.2. Patient Demographics and Tumor Characteristics

#### 3.2.1. IDH-Wildtype Glioblastoma

The average of patients presenting with IDH-wildtype glioblastoma was 59.6 years (SD: 5.6, Range: 49.5–67.0) with 60.8% of pooled patients being male. Tumors were most commonly located in the thalamus (33.3%) followed by the frontoparietal region (19.3%) and corpus callosum (14.9%) (Supplementary Table S1). Just under 10% were categorized as “lobar” without specifying the particular lobe in question. Less than 1% of tumors were located in the parietooccipital and occipital regions. Tumors lateralized to the left in 53.4%, to the right in 30.7%, and were bilateral in 12.4%. The average preoperative tumor volume was 18.1 cm^3^ (SD: 9.23, Range: 9.3–41.0), and 16.8% of tumors exhibited MGMT methylation status. The initial Karnofsky performance scale (KPS) score was 80.6 on average (SD: 3.5, Range: 76.7–85.0).

#### 3.2.2. IDH-Mutant Astrocytoma

The average of patients presenting with IDH-mutant astrocytoma was 38.6 years (SD: 6.3, Range: 33.5–51.0), roughly 60% of patients were male. About 44% of the IDH-mutant astrocytoma were located in the frontoparietal region, 16% were in the thalamus and temporal lobe, 11% were located in the insula, while the remaining 5.6% were either in the corpus callosum or occipital lobe. Additionally, the average preoperative tumor volume was 13.4 cm^3^ (SD: 13.4, Range: 3.1–23.7) with similar lateralization to the right (44.8%) and left (55.2%) hemispheres. Initial KPS was 81.7 on average (SD: 7.6, Range: 75.0–90.0). IDH status was not explicitly reported for 25% of the astrocytoma included in the analysis.

### 3.3. Postoperative Outcomes

#### 3.3.1. IDH-Wildtype Glioblastoma

LITT in IDH-wildtype GBM provided an average extent of ablation (EOA) of 94.8% (SD: 6.1, Range: 87.7–98.6), with tumor progression reported in 50.3% at an average follow-up of 12.11 months and median follow-up of 10.33 months. Average postoperative KPS was 76.5 (SD: 14.2, Range: 63.3–93.3), with a pooled neurologic complication rate of 10.3% and pooled non-neurologic complication rate of 4.8% (Table 2). Neurologic complications included transient aphasia (16.7%), non-specified neurologic deficits (4.5%), hemiparesis (1.3%), and seizure (0.2%). Non-neurologic complications included meningitis (16.7%), deep vein thrombosis (DVT) (1.6%), pulmonary embolism (1.4%), cerebral edema (1.3%), and hydrocephalus (0.4%). There were insufficient data to analyze the pooled mortality rate. The average overall survival and progression-free survival rates were 9.3 months (SD: 1.5, Range: 7.1–11.4) and 4.8 months (SD: 2.1, Range: 2.0–7.9), respectively.

#### 3.3.2. IDH-Mutant Astrocytoma

Patients receiving LITT for IDH-mutant astrocytoma had an average EOA of 84.6% (SD: 4.1, Range: 81.7–87.5). Tumor progression rate was not determined due to insufficient data. The pooled overall neurologic complication rate was 33.0% and the non-neurologic complication rate was 8.3% at an average follow-up of 18.46 months and median follow-up of 13.93 months. Neurologic complications included seizure (14.9%), motor deficit (12.8%), non-specified neurologic deficits (8.8%), and hemiparesis (3.6%). Non-neurologic complications included cerebral edema (12.4%) and wound infections (6.9%). Postoperative mortality was not calculated for IDH-mutant astrocytoma due to insufficient data.

### 3.4. Meta-Regression Analysis

Meta-regression analysis was performed between all preoperative characteristics and postoperative outcomes of interest. While no significant correlation was found between IDH-wildtype GBM and IDH-mutant astrocytoma cohorts for EOA, tumor progression, complication rates, tumor volume, or demographic characteristics, a significant negative correlation was noted in three studies between postoperative KPS and tumor progression (β = −0.07, *p* < 0.0001). Tumor progression was also negatively correlated with MGMT methylation (β = −33.7, *p* < 0.0001).

**Figure 2:**
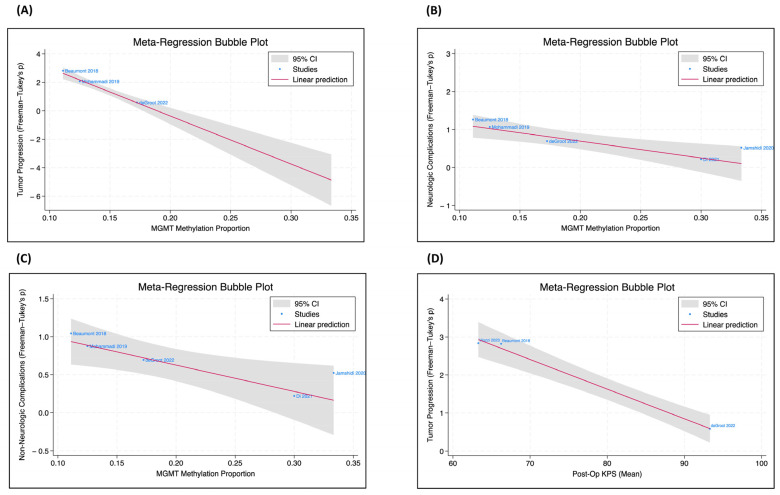
MGMT methylation status, tumor progression, complications, and post-op KPS. The meta-regression bubble plots, only included for the IDH-wildtype GBM subgroup, were utilized to evaluate the correlation coefficient between two patient parameters [16,57,60,63,66,69]. (**A**) There is a significant negative correlation between the proportion of MGMT methylation and tumor progression (*p* < 0.001). (**B**) There is a significant negative relationship between the proportion of MGMT methylation and neurologic complications (*p* = 0.002). (**C**) There is a significant negative relationship between the proportion of MGMT methylation and non-neurologic complications (*p* = 0.015). (**D**) There is a significant negative relationship between postoperative KPS and tumor progression (*p* < 0.001).

Meta-regression analysis within the IDH-wildtype GBM cohort also revealed that MGMT methylated tumors were significantly less likely to have both neurologic (β = −4.4, *p* < 0.01) and non-neurologic complications (β = −3.5, *p* < 0.05) (Figure 2).

### 3.5. Study Heterogeneity

The forest plot analysis reveals the neurologic and non-neurologic complication rates and heterogeneity for the IDH-wildtype GBM and IDH-mutant astrocytoma cohorts (Supplementary Figure S1). Based on the heterogeneity tests, neurologic complications in the IDH-mutant astrocytoma (τ^2^ = 0.24, I^2^ = 47.39%) and the non-neurologic complications in the IDH-wildtype GBM group (τ^2^ = 0.06, I^2^ = 41.10%) demonstrated moderate heterogeneity. Within the IDH-wildtype GBM cohort, the studies reporting neurologic complications demonstrated high heterogeneity (τ^2^ = 0.34, I^2^ = 80.09%). The studies that included non-neurologic complications in the IDH-mutant astrocytoma group were relatively homogenous (τ^2^ = 0.00, I^2^ = 0.00%). Significant cross-study heterogeneity was present in all subgroups except for the non-neurologic complications in the IDH-mutant astrocytoma cohort, which was insignificant (*p* = 0.13).

### 3.6. Adjuvant Therapy in Combination with LITT

We reviewed adjuvant therapies (ATs) used post-LITT among the included studies. Temozolomide, dexamethasone, bevacizumab, lomustine, chemotherapy, tumor treating fields, and pembrolizumab with and without radiation therapy were the reported ATs used post-LITT (Supplementary Table S2). The most commonly occurring AT reported in this study is chemoradiation in the form of temozolomide + radiotherapy (46.5%), as seen in the IDH-wildtype GBM cohort. Although close to 50% of the IDH-wildtype GBM cohort reported the use of ATs post-LITT, just 18% of the IDH-mutant astrocytoma cohort reported receiving any. Overall, the included articles report that the usage of ATs resulted in a higher PFS and OS on average [16,60].

### 3.7. Publication Bias

Publication bias was utilized to evaluate the variance in reporting of LITT efficacy through pooled rates of neurologic and non-neurologic outcomes. Egger’s test did not reveal statistically significant publication bias for non-neurologic complications in either the IDH-wildtype GBM or IDH-mutant astrocytoma cohorts. However, significant publication bias was found to influence the reporting of neurologic complications in studies that evaluated LITT for IDH-mutant astrocytoma (β = −6.2, *p* < 0.01) (Supplementary Figure S2). Specifically, sample size was found to influence significance level, with publication bias against smaller studies.

## 4. Discussion

To the best of our knowledge, this is the first meta-analysis since the 2021 WHO CNS5 guidelines to evaluate the outcomes of LITT as initial therapy in both primary IDH-wildtype GBM and IDH-mutant astrocytoma. Currently, LITT is often used in cases of deep-seated lesions, when there are contraindications to radiotherapy or resection, proximity to eloquent regions, or in cases where patients are particularly vulnerable to wound complications [70,71,72]. In the past, LITT has been shown to reduce postoperative complications while maintaining high ablation rates [69,73]. This study summarizes the current relevant literature on LITT and discusses its viability as an upfront treatment option in the management of primary IDH-wildtype GBM and IDH-mutant astrocytoma.

### 4.1. Survival and Function

The pooled PFS and OS rates for the IDH-wildtype GBM subgroup were 5 and 9 months, respectively. Prior studies have reported similar survival data following surgical resection with PFS and OS of 5 months and 10 months, respectively [25,74,75]. However, it is difficult to place these survival rates in the context of the current literature as many studies combined IDH-wildtype GBM with IDH-mutant astrocytoma when reporting survival rates. Given this heterogeneity in reporting, survival rates in these cohorts are highly variable. One example of this was a 2016 meta-analysis by Ivan et al. in which grade 3 astrocytoma were included with IDH-wildtype GBMs and thus OS was reported as higher at 14.2 months [14]. Also, their conclusions were limited by study power given their analysis only included 3 papers. Our study experienced similar limitations in that LITT is often recommended in instances of unresectable tumors presenting with associated edema, thus potentially biasing the patient selection. While we stratified by tumor type, a secondary limitation was that we were unable to do so by tumor grade given limited patient numbers. Future studies should stratify patients by factors including tumor size, depth, and grade to more accurately reflect LITT’s efficacy compared to standard resection.

One factor that was found to improve survival in our analysis was MGMT methylation status. We found that MGMT-methylated tumors were significantly correlated with improved PFS after LITT (*p* < 0.001), which is consistent with prior studies [16,76]. We also found a significant reduction in postoperative complication rates among patients with MGMT methylation status, suggesting that LITT is particularly safe and effective in these tumors.

In patients whose GBM did not progress during the study period, their KPS score significantly improved following LITT, which is consistent with the previous literature [25,77]. Even in those with recurrence, the safety of LITT has been demonstrated by patients with KPS scores that remain stable long after surgery [69]. However, in this study, the pooled average KPS score decreased somewhat in the immediate postoperative period. The extent and severity of this decline was limited by variations in reporting and lack of long-term follow-up.

Overall, our data suggest that LITT may be a safe primary management option and warrants further investigation through randomized controlled studies to better characterize survival outcomes.

### 4.2. Extent of Ablation

The noted EOA for the IDH-wildtype GBM subgroup (94.8%) is similar to previous studies which report a range between 91 and 99% for newly diagnosed tumors [16,26,62,73]. Although preoperative tumor volume was not controlled, the high EOA supports LITT’s ability to achieve maximal safe ablation in a variety of tumor sizes and locations. The average EOA in the IDH-mutant astrocytoma subgroup was 84.6%, similar to the existing literature. For instance, the study by Ivan et al. reported a tumor ablation rate of 82.9% [14]. However, this study had high heterogeneity with regard to tumor types and molecular profiles included [14]. Past studies have indicated that a 78% extent of resection is the threshold for significantly improved overall survival in patients undergoing surgery [33,78,79]. The extent of ablation in both cohorts exceeded this threshold on average, though it is unclear if this percentage should be applied given its original context was in regard to the extent of the resection. Maximization of EOA involves optimizing LITT-associated parameters such as optimal trajectory, integration of intraoperative neuromonitoring, and a risk assessment of lesion-to-tract distance to prevent significant disability in tracts thermally susceptible [48,80]. More prospective studies are needed to address the role of EOA on both survival and postoperative neurologic deficit to determine ideal parameters for these cases.

### 4.3. Postoperative Complications

In our analysis, LITT for IDH-wildtype GBM demonstrated lower postoperative neurologic (10.3%) and non-neurologic (4.8%) complication rates relative to those reported after other treatment modalities noted by the literature. Advancements in LITT have resulted in lower complication rates in comparison to the Viozzi et al. study that reported a rate of 33.7% in newly diagnosed GBM post-LITT [81]. A prospective study by Zetterling et al. examined complications following surgical resection using 5-ALA contrast and found that 41% experienced neurologic deficits postoperatively [82]. In another prospective analysis, Gempt et al. found ischemic lesions following surgery for GBM in 31% of cases, 24% were symptomatic [83]. Similarly, other national outcome studies report high complication rates between 13 and 19% after craniotomy [84,85,86,87,88]. This appears true with regard to patients undergoing hypofractionated radiotherapy as well [78,79]. As reported by Floyd et al., late toxicity effects based on the Radiation Therapy Oncology Group neurotoxicity scores, were reported in 50% of individuals with an average radiation dosage of 50 Gy applied during hypofractionated radiotherapy [79]. Our findings suggest a comparable extent of ablation/resection with lower complication rates in patients with GBM primarily treated with LITT as opposed to surgery or radiation.

Interestingly, we found neurologic complication rates that were three times as high for IDH-mutant astrocytoma when compared to GBM (33% vs. 10%). However, the significance of this is difficult to interpret given the low sample size in the IDH-mutant astrocytoma cohort and the variability in IDH-mutant astrocytoma grades included [89,90]. Aside from neurologic complications in the IDH-mutant astrocytoma cohort, all other complication rates for both tumor subtypes were similar to the neurologic rates presented in the literature ranging from 2.17% to 7.14% [80,91,92]. Given that LITT is often used for deep-seated tumors located near eloquent structures, these relatively low complication rates are reassuring. While LITT is a minimally-invasive technique, technical complications may arise, such as hyperthermic deposition, catheter placement, and malfunctioning of the cooling system [93]. Institutional recognition and reporting of these complications is necessary for improving the software and hardware involved.

### 4.4. Adjuvant Therapies

Currently, adjuvant chemotherapy is recommended along with maximal surgical resection. However, 30% of GBM cases are considered inoperable [94]. The ability of LITT to target these tumors in concert with AT to augment the penetration of the peritumoral BBB highlights its utility in multimodal management [32]. Leuthardt et al. reported increased peritumoral BBB permeability from weeks 1 to 2 and 4 to 6, highlighting the ability of LITT to enhance drug delivery to allow for the use of certain adjuvant therapies [37]. Although we were unable to run a meta-analysis for multimodal therapies in combination with LITT due to lack of enough data points and inconsistent reporting, our study was able to systematically review and characterize post-LITT adjuvant therapies from the few studies that did not include them. The most commonly occurring AT reported in this study is chemoradiation in the form of temozolomide + radiotherapy (46.5%), while the use of the immunotherapeutic pembrolizumab alone was the least utilized AT in the GBM cohort. Based on our findings, adjuvant therapies appear to provide improved survival outcomes after LITT, and this is reflected in the literature. For example, one multimodal study of LITT and chemoradiation for newly diagnosed IDH-wildtype GBM found an OS of 16 months and PFS of 12 months with combined treatment versus an OS of 10 months and PFS of 6 months after LITT alone [16]. Currently, the active expedited laser interstitial thermal therapy and chemoradiation clinical trial (NCT02970448) is evaluating the feasibility of LITT with standard concurrent radiotherapy and temozolomide in newly diagnosed high-grade gliomas [95]. In the GBM cohort, two individuals (1.1%) received tumor-treating field therapy, which delivers low intermediate frequency electric fields to selectively remove proliferative cells, increase survival outcomes, and reduce recurrence [56]. While the adjuvant immunotherapeutic pembrolizumab was administered in only one GBM patient in our cohort (0.5%), it has been shown to increase peripheral immune cell recruitment and immune response in GBM [16,96]. Although a current phase I/II study (NCT02311582) is evaluating the in situ vaccination effect of LITT using PD-L1 inhibitor pembrolizumab at varying dosages for recurrent tumors, similar trials need to be conducted for primary CNS tumors [97]. Prospective studies are needed to evaluate the potential of adjuvant LITT to improve drug delivery, radiotherapy efficacy, and survival.

### 4.5. Limitations and Future Steps

This meta-analysis had inherent limitations due to nonuniform reporting of demographics and outcomes amongst the included studies, as well as overall diminished sample size, especially in the IDH-mutant astrocytoma cohort. The lack of standardized technical parameters in LITT, such as ablation duration or temperature and operator experience, resulted in variability between studies. Additionally, the methodology of EOA measurement was inconsistently reported by studies, often being limited to MRI utilized post-procedurally. We utilized random-effects modeling in the meta-analyses to account for interstudy heterogeneity. Due to limited sample sizes and study count, tumor types such as meningiomas and oligodendrogliomas were not included. As different tumors are characterized by varying growth patterns and invasion, we performed subgroup analyses by tumor type and excluded metastatic and recurrent tumors. Within the IDH-mutant astrocytoma cohort, we did not differentiate by tumor grade as we were limited by the number of studies and heterogeneity in reporting. In terms of genetic characteristics, IDH1 mutation and MGMT methylation could not be extracted for studies in which these statuses were not reported for the entire study cohort. Consequently, any IDH-wildtype tumors reported in the studies, but unidentified for IDH mutation, could not be reclassified based on the WHO CNS5 classification. This highlights the necessity of future research to report all pertinent genetic characteristics, especially due to potential revisions to the WHO CNS5 stratification for tumor type. Thus, future meta-analyses can reclassify such tumors, offering insights into survival outcomes based on the most current tumor classification criteria. Further, we were not able to control for follow-up time in our study, which resulted in variability of the reported outcomes. Only the IDH-mutant astrocytoma group appeared to have significant publication bias in association with neurologic outcomes, which is likely due to the small sample size of this cohort. Nonetheless, this is the first proportional meta-analysis exploring the utility of LITT in the management of primary CNS tumors following the updated WHO CNS5 stratification by tumor type. In the evaluation of treatment modalities, this study assumes EOR and EOA are comparable in terms of tumor removal, modeling after previous retrospective studies [77,98]. Adoption of a more uniform procedure and data reporting may aid in future analysis of the efficacy of adjuvant therapies post-LITT. Future prospective studies are required to validate these results and for direct comparison of LITT to other forms of treatment. Large-scale studies may also aid in the evaluation of differences in outcome, wavelength, cooling modality, and laser power between the three FDA-approved LITT systems Monteris NeuroBlate^®^, Medtronic Visualase^™^, and ClearPoint Prism^®^ in optimizing their parameters [12,48,70,99,100].

## 5. Conclusions

We present the first meta-analysis of clinical outcomes following LITT since the publication of the 2021 WHO CNS5 guidelines for two of the most common primary CNS tumor types. We found that LITT for IDH-wildtype GBM provides acceptable EOA with similar to reduced complication rates when compared to surgical resection. Our investigation into IDH-mutant astrocytoma demonstrated slightly lower EOA on average with higher complications, though it is difficult to draw any robust conclusions from this cohort given its extremely limited size of just 21 patients. However, these findings combined with the unique ability of LITT to enhance drug delivery and radiotherapy efficacy suggests its utility as an adjuvant treatment modality bears further investigation. Larger prospective studies are needed to establish clear recommendations for LITT as a primary treatment option for these challenging lesions.

## Figures and Tables

**Table 1:** Patient Demographics and Characteristics.

Study	Tumor Grade	Patients (N)	Mean Age (Years)	Males (%)	Mean KPS Pre-Op	Mean Tumor Volume (cc)
**Astrocytoma**						
Borghei-Razavi 2018 [52]	1	2	37	50	-	3
Johnson 2022 [53] *	3	2	37	100	90	-
Johnson 2022 [53] *	2	3	34	33	80	-
Kahn 1994 [54]	2	6	51	-	-	-
Kunesch 2003 [55]	2	5	35	60	-	-
Murayi 2020 [56] †	3	2	39	50	75	24
**Glioblastoma**						
Beaumont 2018 [57]	4	9	55	78	80	23
Dabecco 2021 [58]	4	4	50	-	-	-
Daggubati 2023 [59]	4	9	-	-	-	-
deGroot 2022 [16]	4	29	63	69	-	-
Di 2021 [60]	4	20	63	60	85	41
Hajtovic 2020 [61]	4	2	67	100	-	11
Hawasli 2013 [62]	4	6	55	67	-	17
Jamshidi 2020 [63]	4	3	64	67	83	15
Kamath 2019 [46]	4	23	-	-	-	-
Maraka 2018 [64]	4	4	-	-	-	-
Merenzon 2023 [65]	4	5	66	-	78	-
Mohammadi 2019 [66]	4	24	54	50	-	9
Muir 2022 [67]	4	20	-	60	84	15
Murayi 2020 [56] †	4	9	55	56	77	11
Thomas 2016 [68]	4	8	61	-	-	22
Viozzi 2023 [69]	4	10	63	30	77	16

The patient demographic data and characteristics from the 22 included studies differentiated by tumor type (GBM or astrocytoma). *: Johnson et al. [53] is reported twice for independent astrocytoma grades, the first representing two patients with astrocytoma grade 2 and the second with three patients with astrocytoma grade 3. †: Murayi et al. [56] is reported twice for two independent tumor type cohorts: the first reports astrocytoma and the second reports only GBM patients from the same study. *IDH1: Isocitrate dehydrogenase 1*; *MGMT: O(6)-methylguanine-DNA methyltransferase; KPS: Karnofsky performance status.*

**Table 2:** Patient Postoperative Outcomes.

Study ID	Follow-Up (Months)	Mean EOA (%)	Mean OS (Months)	Mean PFS (Months)	Mean KPS Post-Op
**Astrocytoma**					
Borghei-Razavi 2018 [52]	14	-	-	-	-
Johnson 2022 [53] *	7	88	-	-	-
Johnson 2022 [53] *	36	82	-	-	-
Kahn 1994 [54]	17	-	-	-	-
Kunesch 2003 [55]	18	-	-	-	-
Murayi 2020 [56] †	-	-	-	-	-
**Glioblastoma**					
Beaumont 2018 [57]	-	-	10	4	66
Dabecco 2021 [58]	-	98	-	-	-
Daggubati 2023 [59]	-	-	-	-	-
deGroot 2022 [16]	-	-	10	6	93
Di 2021 [60]	10	88	-	8	-
Hajtovic 2020 [61]	-	-	-	-	-
Hawasli 2013 [62]	5	-	-	-	-
Jamshidi 2020 [63]	6	99	-	4	83
Kamath 2019 [46]	11	-	11	6	-
Maraka 2018 [64]	-	-	-	-	-
Merenzon 2023 [65]	31	-	9	-	-
Mohammadi 2019 [66]	14	-	-	-	-
Muir 2022 [67]	18	-	-	-	-
Murayi 2020 [56] †	-	-	7	-	-
Thomas 2016 [68]	-	-	8	2	-
Viozzi 2023 [69]	3	-	-	-	63

The patient postoperative outcomes from the 22 included studies differentiated by tumor type (GBM or astrocytoma). *: Johnson et al. [53] is reported twice for independent astrocytoma grades, the first representing two patients with astrocytoma grade 2 and the second with three patients with astrocytoma grade 3. †: Murayi et al. [56] is reported twice for two independent tumor type cohorts: the first reports astrocytoma and the second reports only GBM patients from the same study. Note: *The postoperative outcomes are reported from the patient’s last follow-up at the time of each study. Mean KPS postoperative scores were recorded at 1 month follow-up in Jamshidi et al. and Beaumont et al. while at 3 months follow-up for deGroot et al. and Viozzi et al. EOA: Extent of ablation; OS: Overall survival; PFS: Progression-free survival; KPS: Karnofsky performance status.*

## Data Availability

The data presented in this study are available in this article and Supplementary Materials.

## Supplementary Materials

The supporting information can be downloaded at https://www.mdpi.com/article/10.3390/cancers16112131/s1, Table S1: Tumor Location; Table S2: Adjuvant Therapy in Combination with LITT; Figure S1: Forest Plots for Type of Complication and Tumor; Figure S2: Egger’s Test for Publication Bias.

## References

[1] Torp S.H., Solheim O., Skjulsvik A.J. (2022). The WHO 2021 Classification of Central Nervous System Tumours: A Practical Update on What Neurosurgeons Need to Know—A Minireview. Acta Neurochir..

[2] Horbinski C., Berger T., Packer R.J., Wen P.Y. (2022). Clinical Implications of the 2021 Edition of the WHO Classification of Central Nervous System Tumours. Nat. Rev. Neurol..

[3] Louis D.N., Perry A., Wesseling P., Brat D.J., Cree I.A., Figarella-Branger D., Hawkins C., Ng H.K., Pfister S.M., Reifenberger G. (2021). The 2021 WHO Classification of Tumors of the Central Nervous System: A Summary. Neuro-Oncology.

[4] McNamara C., Mankad K., Thust S., Dixon L., Limback-Stanic C., D’Arco F., Jacques T.S., Löbel U. (2022). 2021 WHO Classification of Tumours of the Central Nervous System: A Review for the Neuroradiologist. Neuroradiology.

[5] Wen P.Y., Packer R.J. (2021). The 2021 WHO Classification of Tumors of the Central Nervous System: Clinical Implications. Neuro-Oncology.

[6] Johnson D.R., Giannini C., Vaubel R.A., Morris J.M., Eckel L.J., Kaufmann T.J., Guerin J.B. (2022). A Radiologist’s Guide to the 2021 WHO Central Nervous System Tumor Classification: Part I—Key Concepts and the Spectrum of Diffuse Gliomas. Radiology.

[7] Gritsch S., Batchelor T.T., Gonzalez Castro L.N. (2022). Diagnostic, Therapeutic, and Prognostic Implications of the 2021 World Health Organization Classification of Tumors of the Central Nervous System. Cancer.

[8] Lakhani D.A., Sabsevitz D.S., Chaichana K.L., Quiñones-Hinojosa A., Middlebrooks E.H. (2023). Current State of Functional MRI in the Presurgical Planning of Brain Tumors. Radiol. Imaging Cancer.

[9] Shah S., Ivey N., Matur A., Andaluz N. (2023). Intraoperative Fluorophores: An Update on 5-Aminolevulinic Acid and Sodium Fluorescein in Resection of Tumors of the Central Nervous System and Metastatic Lesions-A Systematic Review and Meta-Analysis. Tomography.

[10] Osborn A.G., Louis D.N., Poussaint T.Y., Linscott L.L., Salzman K.L. (2022). The 2021 World Health Organization Classification of Tumors of the Central Nervous System: What Neuroradiologists Need to Know. Am. J. Neuroradiol..

[11] Xue F., Chen T., Sun H. (2018). Postoperative Outcomes of Magnetic Resonance Imaging (MRI)-Guided Laser Interstitial Thermal Therapy (LITT) in the Treatment of Drug-Resistant Epilepsy: A Meta-Analysis. Med. Sci. Monit..

[12] Salem U., Kumar V.A., Madewell J.E., Schomer D.F., De Almeida Bastos D.C., Zinn P.O., Weinberg J.S., Rao G., Prabhu S.S., Colen R.R. (2019). Neurosurgical Applications of MRI Guided Laser Interstitial Thermal Therapy (LITT). Cancer Imaging.

[13] Hegi M.E., Diserens A.-C., Gorlia T., Hamou M.-F., de Tribolet N., Weller M., Kros J.M., Hainfellner J.A., Mason W., Mariani L. (2005). MGMT Gene Silencing and Benefit from Temozolomide in Glioblastoma. N. Engl. J. Med..

[14] Ivan M.E., Mohammadi A.M., De Deugd N., Reyes J., Rodriguez G., Shah A., Barnett G.H., Komotar R.J. (2016). Laser Ablation of Newly Diagnosed Malignant Gliomas: A Meta-Analysis. Neurosurgery.

[15] Chen C., Lee I., Tatsui C., Elder T., Sloan A.E. (2021). Laser Interstitial Thermotherapy (LITT) for the Treatment of Tumors of the Brain and Spine: A Brief Review. J. Neurooncol..

[16] de Groot J.F., Kim A.H., Prabhu S., Rao G., Laxton A.W., Fecci P.E., O’Brien B.J., Sloan A., Chiang V., Tatter S.B. (2022). Efficacy of Laser Interstitial Thermal Therapy (LITT) for Newly Diagnosed and Recurrent IDH Wild-Type Glioblastoma. Neuro-Oncol. Adv..

[17] Patel T.R., Chiang V.L.S. (2014). Laser Interstitial Thermal Therapy for Treatment of Post-Radiosurgery Tumor Recurrence and Radiation Necrosis. Photonics Lasers Med..

[18] Lerner E.C., Edwards R.M., Wilkinson D.S., Fecci P.E. (2022). Laser Ablation: Heating up the Anti-Tumor Response in the Intracranial Compartment. Adv. Drug Deliv. Rev..

[19] Fabiano A.J., Alberico R.A. (2014). Laser-Interstitial Thermal Therapy for Refractory Cerebral Edema from Post-Radiosurgery Metastasis. World Neurosurg..

[20] Bown S.G. (1983). Phototherapy of Tumors. World J. Surg..

[21] Aizer A.A., Lamba N., Ahluwalia M.S., Aldape K., Boire A., Brastianos P.K., Brown P.D., Camidge D.R., Chiang V.L., Davies M.A. (2022). Brain Metastases: A Society for Neuro-Oncology (SNO) Consensus Review on Current Management and Future Directions. Neuro-Oncology.

[22] Desclides M., Ozenne V., Bour P., Faller T., Machinet G., Pierre C., Chemouny S., Quesson B. (2023). Real-Time Automatic Temperature Regulation during In Vivo MRI-Guided Laser-Induced Thermotherapy (MR-LITT). Sci. Rep..

[23] Buckley R.T., Wang A.C., Miller J.W., Novotny E.J., Ojemann J.G. (2016). Stereotactic Laser Ablation for Hypothalamic and Deep Intraventricular Lesions. Neurosurg. Focus.

[24] Medvid R., Ruiz A., Komotar R.J., Jagid J.R., Ivan M.E., Quencer R.M., Desai M.B. (2015). Current Applications of MRI-Guided Laser Interstitial Thermal Therapy in the Treatment of Brain Neoplasms and Epilepsy: A Radiologic and Neurosurgical Overview. Am. J. Neuroradiol..

[25] Kim A.H., Tatter S., Rao G., Prabhu S., Chen C., Fecci P., Chiang V., Smith K., Williams B.J., Mohammadi A.M. (2020). Laser Ablation of Abnormal Neurological Tissue Using Robotic NeuroBlate System (LAANTERN): 12-Month Outcomes and Quality of Life After Brain Tumor Ablation. Neurosurgery.

[26] Shah A.H., Semonche A., Eichberg D.G., Borowy V., Luther E., Sarkiss C.A., Morell A., Mahavadi A.K., Ivan M.E., Komotar R.J. (2020). The Role of Laser Interstitial Thermal Therapy in Surgical Neuro-Oncology: Series of 100 Consecutive Patients. Neurosurgery.

[27] Boop S., Bonda D., Randle S., Leary S., Vitanza N., Crotty E., Novotny E., Friedman S., Ellenbogen R.G., Durfy S. (2023). A Comparison of Clinical Outcomes for Subependymal Giant Cell Astrocytomas Treated with Laser Interstitial Thermal Therapy, Open Surgical Resection, and mTOR Inhibitors. Pediatr. Neurosurg..

[28] Yudkoff C., Mahtabfar A., Piper K., Judy K. (2022). Safety and Efficacy of Salvage Therapy with Laser Interstitial Thermal Therapy for Malignant Meningioma Refractory to Cesium-131 Brachytherapy: Illustrative Case. J. Neurosurg. Case Lessons.

[29] Ali S.C., Basil G.W., Diaz R.J., Komotar R.J. (2018). The Safety of Bevacizumab Administered Shortly after Laser Interstitial Thermal Therapy in Glioblastoma: A Case Series. World Neurosurg..

[30] Butt O.H., Zhou A.Y., Huang J., Leidig W.A., Silberstein A.E., Chheda M.G., Johanns T.M., Ansstas G., Liu J., Talcott G. (2022). Corrigendum to: A Phase II Study of Laser Interstitial Thermal Therapy Combined with Doxorubicin in Patients with Recurrent Glioblastoma. Neuro-Oncol. Adv..

[31] Fomchenko E.I., Leelatian N., Darbinyan A., Huttner A.J., Chiang V.L. (2022). Histological Changes Associated with Laser Interstitial Thermal Therapy for Radiation Necrosis: Illustrative Cases. J. Neurosurg. Case Lessons.

[32] Salehi A., Paturu M.R., Patel B., Cain M.D., Mahlokozera T., Yang A.B., Lin T.-H., Leuthardt E.C., Yano H., Song S.-K. (2020). Therapeutic Enhancement of Blood-Brain and Blood-Tumor Barriers Permeability by Laser Interstitial Thermal Therapy. Neuro-Oncol. Adv..

[33] Balança B., Meiller A., Bezin L., Dreier J.P., Marinesco S., Lieutaud T. (2017). Altered Hypermetabolic Response to Cortical Spreading Depolarizations after Traumatic Brain Injury in Rats. J. Cereb. Blood Flow Metab..

[34] Hu L.S., Brat D.J., Bloch O., Ramkissoon S., Lesser G.J. (2020). The Practical Application of Emerging Technologies Influencing the Diagnosis and Care of Patients with Primary Brain Tumors. Am. Soc. Clin. Oncol. Educ. Book.

[35] Sabel M., Rommel F., Kondakci M., Gorol M., Willers R., Bilzer T. (2003). Locoregional Opening of the Rodent Blood-Brain Barrier for Paclitaxel Using Nd:YAG Laser-Induced Thermo Therapy: A New Concept of Adjuvant Glioma Therapy?. Lasers Surg. Med..

[36] Pardridge W.M. (2005). The Blood-Brain Barrier: Bottleneck in Brain Drug Development. NeuroRX.

[37] Leuthardt E.C., Duan C., Kim M.J., Campian J.L., Kim A.H., Miller-Thomas M.M., Shimony J.S., Tran D.D. (2016). Hyperthermic Laser Ablation of Recurrent Glioblastoma Leads to Temporary Disruption of the Peritumoral Blood Brain Barrier. PLoS ONE.

[38] Demeule M., Régina A., Jodoin J., Laplante A., Dagenais C., Berthelet F., Moghrabi A., Béliveau R. (2002). Drug Transport to the Brain: Key Roles for the Efflux Pump P-Glycoprotein in the Blood-Brain Barrier. Vascul. Pharmacol..

[39] Sanders S., Debinski W. (2020). Challenges to Successful Implementation of the Immune Checkpoint Inhibitors for Treatment of Glioblastoma. Int. J. Mol. Sci..

[40] Kiyatkin E.A., Sharma H.S. (2009). Permeability of the Blood-Brain Barrier Depends on Brain Temperature. Neuroscience.

[41] Hong C.S., Deng D., Vera A., Chiang V.L. (2019). Laser-Interstitial Thermal Therapy Compared to Craniotomy for Treatment of Radiation Necrosis or Recurrent Tumor in Brain Metastases Failing Radiosurgery. J. Neurooncol..

[42] Schwalb A.M., Srinivasan E.S., Fecci P.E. (2022). Commentary: Laser Interstitial Thermal Therapy for First-Line Treatment of Surgically Accessible Recurrent Glioblastoma: Outcomes Compared with a Surgical Cohort. Neurosurgery.

[43] Voigt J.D., Barnett G. (2016). The Value of Using a Brain Laser Interstitial Thermal Therapy (LITT) System in Patients Presenting with High Grade Gliomas Where Maximal Safe Resection May Not Be Feasible. Cost Eff. Resour. Alloc..

[44] Salehi A., Kamath A.A., Leuthardt E.C., Kim A.H. (2018). Management of Intracranial Metastatic Disease with Laser Interstitial Thermal Therapy. Front. Oncol..

[45] Patel N.V., Mian M., Stafford R.J., Nahed B.V., Willie J.T., Gross R.E., Danish S.F. (2016). Laser Interstitial Thermal Therapy Technology, Physics of Magnetic Resonance Imaging Thermometry, and Technical Considerations for Proper Catheter Placement During Magnetic Resonance Imaging–Guided Laser Interstitial Thermal Therapy. Neurosurgery.

[46] Kamath A.A., Friedman D.D., Akbari S.H.A., Kim A.H., Tao Y., Luo J., Leuthardt E.C. (2019). Glioblastoma Treated with Magnetic Resonance Imaging-Guided Laser Interstitial Thermal Therapy: Safety, Efficacy, and Outcomes. Neurosurgery.

[47] Page M.J., McKenzie J.E., Bossuyt P.M., Boutron I., Hoffmann T.C., Mulrow C.D., Shamseer L., Tetzlaff J.M., Akl E.A., Brennan S.E. (2021). The PRISMA 2020 Statement: An Updated Guideline for Reporting Systematic Reviews. BMJ.

[48] Patel B., Kim A.H. (2020). Laser Interstitial Thermal Therapy. Mo Med..

[49] StataCorp (2023). Stata Statistical Software: Release 18.

[50] Cai S., Zhou J., Pan J. (2021). Estimating the Sample Mean and Standard Deviation from Order Statistics and Sample Size in Meta-Analysis. Stat. Methods Med. Res..

[51] McGrath S., Katzenschlager S., Zimmer A.J., Seitel A., Steele R., Benedetti A. (2023). Standard Error Estimation in Meta-Analysis of Studies Reporting Medians. Stat. Methods Med. Res..

[52] Borghei-Razavi H., Koech H., Sharma M., Krivosheya D., Lee B.S., Barnett G.H., Mohammadi A.M. (2018). Laser Interstitial Thermal Therapy for Posterior Fossa Lesions: An Initial Experience. World Neurosurg..

[53] Johnson G.W., Han R.H., Smyth M.D., Leuthardt E.C., Kim A.H. (2022). Laser Interstitial Thermal Therapy in Grade 2/3 IDH1/2 Mutant Gliomas: A Preliminary Report and Literature Review. Curr. Oncol..

[54] Kahn T., Bettag M., Ulrich F., Schwarzmaier H.J., Schober R., Fürst G., Mödder U. (1994). MRI-Guided Laser-Induced Interstitial Thermotherapy of Cerebral Neoplasms. J. Comput. Assist. Tomogr..

[55] Kunesch E., Classen J., Bettag M., Kahn T., Ulrich F., Bock W.J., Freund H.J., Seitz R.J. (2003). Representational Cortical Plasticity Associated with Brain Tumours: Evidence from Laser-Induced Interstitial Thermotherapy. Acta Neurol. Scand..

[56] Murayi R., Borghei-Razavi H., Barnett G.H., Mohammadi A.M. (2020). Laser Interstitial Thermal Therapy in the Treatment of Thalamic Brain Tumors: A Case Series. Oper. Neurosurg..

[57] Beaumont T.L., Mohammadi A.M., Kim A.H., Barnett G.H., Leuthardt E.C. (2018). Magnetic Resonance Imaging-Guided Laser Interstitial Thermal Therapy for Glioblastoma of the Corpus Callosum. Neurosurgery.

[58] Dabecco R., Gigliotti M.J., Mao G., Myers D., Xu L., Lee P., Ranjan T., Aziz K., Yu A. (2021). Laser Interstitial Thermal Therapy (LITT) for Intracranial Lesions: A Single-Institutional Series, Outcomes, and Review of the Literature. Br. J. Neurosurg..

[59] Daggubati L.C., Ramos-Fresnedo A., Merenzon M.A., Bhatia S., Morell A.A., Berry K.M., Chandar J., Shah A.H., Komotar R.J., Ivan M.E. (2023). Bilateral Laser Interstitial Thermal Therapy for Butterfly Gliomas Compared with Needle Biopsy: A Preliminary Survival Study. Oper. Neurosurg..

[60] Di L., Wang C.P., Shah A.H., Eichberg D.G., Semonche A.M., Sanjurjo A.D., Luther E.M., Jermakowicz W.J., Komotar R.J., Ivan M.E. (2021). A Cohort Study on Prognostic Factors for Laser Interstitial Thermal Therapy Success in Newly Diagnosed Glioblastoma. Neurosurgery.

[61] Hajtovic S., Mogilner A., Ard J., Gautreaux J.E., Britton H., Fatterpekar G., Young M.G., Placantonakis D.G. (2020). Awake Laser Ablation for Patients with Tumors in Eloquent Brain Areas: Operative Technique and Case Series. Cureus.

[62] Hawasli A.H., Bagade S., Shimony J.S., Miller-Thomas M., Leuthardt E.C. (2013). Magnetic Resonance Imaging-Guided Focused Laser Interstitial Thermal Therapy for Intracranial Lesions: Single-Institution Series. Neurosurgery.

[63] Jamshidi A.M., Eichberg D.G., Komotar R.J., Ivan M. (2020). Safety Analysis of Bilateral Laser Interstitial Thermal Therapy for Treatment of Butterfly Glioma. World Neurosurg..

[64] Maraka S., Asmaro K., Walbert T., Lee I. (2018). Cerebral Edema Induced by Laser Interstitial Thermal Therapy and Radiotherapy in Close Succession in Patients with Brain Tumor. Lasers Surg. Med..

[65] Merenzon M.A., Patel N.V., Morell A.A., Marcó Del Pont F., Moll J.M., Komotar R.J., Ivan M.E. (2023). Newly Diagnosed Adult Basal Ganglia Gliomas Treated with Laser Interstitial Thermal Therapy: A Comparative Cohort with Needle Biopsy. Oper. Neurosurg..

[66] Mohammadi A.M., Sharma M., Beaumont T.L., Juarez K.O., Kemeny H., Dechant C., Seas A., Sarmey N., Lee B.S., Jia X. (2019). Upfront Magnetic Resonance Imaging-Guided Stereotactic Laser-Ablation in Newly Diagnosed Glioblastoma: A Multicenter Review of Survival Outcomes Compared to a Matched Cohort of Biopsy-Only Patients. Neurosurgery.

[67] Muir M., Patel R., Traylor J.I., de Almeida Bastos D.C., Kamiya C., Li J., Rao G., Prabhu S.S. (2022). Laser Interstitial Thermal Therapy for Newly Diagnosed Glioblastoma. Lasers Med. Sci..

[68] Thomas J.G., Rao G., Kew Y., Prabhu S.S. (2016). Laser Interstitial Thermal Therapy for Newly Diagnosed and Recurrent Glioblastoma. Neurosurg. Focus.

[69] Viozzi I., Overduin C.G., Rijpma A., Rovers M.M., Laan M.T. (2023). MR-Guided LITT Therapy in Patients with Primary Irresectable Glioblastoma: A Prospective, Controlled Pilot Study. J. Neurooncol..

[70] Mirza F., Mitha R., Shamim M. (2020). Current Role of Laser Interstitial Thermal Therapy in the Treatment of Intracranial Tumors. Asian J. Neurosurg..

[71] Yu P., Yang Y. (2024). Meta-Analysis of the Impact of Laser Interstitial Hyperthermia on Wound Healing Complications in Brain Tumors. Int. Wound J..

[72] Sabahi M., Bordes S.J., Najera E., Mohammadi A.M., Barnett G.H., Adada B., Borghei-Razavi H. (2022). Laser Interstitial Thermal Therapy for Posterior Fossa Lesions: A Systematic Review and Analysis of Multi-Institutional Outcomes. Cancers.

[73] Wright J., Chugh J., Wright C.H., Alonso F., Hdeib A., Gittleman H., Barnholtz-Sloan J., Sloan A.E. (2016). Laser Interstitial Thermal Therapy Followed by Minimal-Access Transsulcal Resection for the Treatment of Large and Difficult to Access Brain Tumors. Neurosurg. Focus.

[74] AbdelFatah M.A.R., Kotb A., Said M.A., Abouelmaaty E.M.H. (2022). Impact of Extent of Resection of Newly Diagnosed Glioblastomas on Survival: A Meta-Analysis. Egypt. J. Neurosurg..

[75] Kaisman-Elbaz T., Xiao T., Grabowski M.M., Barnett G.H., Mohammadi A.M. (2023). The Impact of Extent of Ablation on Survival of Patients with Newly Diagnosed Glioblastoma Treated with Laser Interstitial Thermal Therapy: A Large Single-Institutional Cohort. Neurosurgery.

[76] Rivera A.L., Pelloski C.E., Gilbert M.R., Colman H., De La Cruz C., Sulman E.P., Bekele B.N., Aldape K.D. (2010). MGMT Promoter Methylation Is Predictive of Response to Radiotherapy and Prognostic in the Absence of Adjuvant Alkylating Chemotherapy for Glioblastoma. Neuro-Oncology.

[77] Mohammadi A.M., Hawasli A.H., Rodriguez A., Schroeder J.L., Laxton A.W., Elson P., Tatter S.B., Barnett G.H., Leuthardt E.C. (2014). The Role of Laser Interstitial Thermal Therapy in Enhancing Progression-Free Survival of Difficult-to-Access High-Grade Gliomas: A Multicenter Study. Cancer Med..

[78] Roa W., Kepka L., Kumar N., Sinaika V., Matiello J., Lomidze D., Hentati D., Guedes De Castro D., Dyttus-Cebulok K., Drodge S. (2015). International Atomic Energy Agency Randomized Phase III Study of Radiation Therapy in Elderly and/or Frail Patients with Newly Diagnosed Glioblastoma Multiforme. J. Clin. Oncol..

[79] Floyd N.S., Woo S.Y., Teh B.S., Prado C., Mai W.-Y., Trask T., Gildenberg P.L., Holoye P., Augspurger M.E., Carpenter L.S. (2004). Hypofractionated Intensity-Modulated Radiotherapy for Primary Glioblastoma Multiforme. Int. J. Radiat. Oncol..

[80] Holste K.G., Orringer D.A. (2020). Laser Interstitial Thermal Therapy. Neuro-Oncol. Adv..

[81] Viozzi I., Guberinic A., Overduin C.G., Rovers M.M., Ter Laan M. (2021). Laser Interstitial Thermal Therapy in Patients with Newly Diagnosed Glioblastoma: A Systematic Review. J. Clin. Med..

[82] Zetterling M., Elf K., Semnic R., Latini F., Engström E.R. (2020). Time Course of Neurological Deficits after Surgery for Primary Brain Tumours. Acta Neurochir..

[83] Gempt J., Förschler A., Buchmann N., Pape H., Ryang Y.-M., Krieg S.M., Zimmer C., Meyer B., Ringel F. (2013). Postoperative Ischemic Changes Following Resection of Newly Diagnosed and Recurrent Gliomas and Their Clinical Relevance: Clinical Article. J. Neurosurg..

[84] Gulati S., Jakola A.S., Nerland U.S., Weber C., Solheim O. (2011). The Risk of Getting Worse: Surgically Acquired Deficits, Perioperative Complications, and Functional Outcomes After Primary Resection of Glioblastoma. World Neurosurg..

[85] Wang W.-L., Aru N., Liu Z., Shen X., Ding Y.-M., Wu S.-J., Qin H.-H., Jin W.-Y. (2019). Prognosis of Patients with Newly Diagnosed Glioblastoma Treated with Molecularly Targeted Drugs Combined with Radiotherapy vs Temozolomide Monotherapy: A Meta-Analysis. Medicine.

[86] Chaichana K.L., Jusue-Torres I., Navarro-Ramirez R., Raza S.M., Pascual-Gallego M., Ibrahim A., Hernandez-Hermann M., Gomez L., Ye X., Weingart J.D. (2014). Establishing Percent Resection and Residual Volume Thresholds Affecting Survival and Recurrence for Patients with Newly Diagnosed Intracranial Glioblastoma. Neuro-Oncology.

[87] Gerritsen J.K.W., Arends L., Klimek M., Dirven C.M.F., Vincent A.J.-P.E. (2019). Impact of Intraoperative Stimulation Mapping on High-Grade Glioma Surgery Outcome: A Meta-Analysis. Acta Neurochir..

[88] Trinh V.T., Davies J.M., Berger M.S. (2015). Surgery for Primary Supratentorial Brain Tumors in the United States, 2000–2009: Effect of Provider and Hospital Caseload on Complication Rates. J. Neurosurg..

[89] Mao H., Li X., Mao W. (2020). Advantages of Gross Total Resection in Patients with Astrocytoma: A Population-based Study. Oncol. Lett..

[90] Wen P.Y., Kesari S. (2008). Malignant Gliomas in Adults. N. Engl. J. Med..

[91] Sakai T., Fujishima I., Sugiyama K., Ryu H., Uemura K. (1992). Interstitial Laserthermia in Neurosurgery. J. Clin. Laser Med. Surg..

[92] Levy A.S., Merenzon M.A., Eatz T., Morell A.A., Eichberg D.G., Bloom M.J., Shah A.H., Komotar R.J., Ivan M.E. (2023). Development of an Enhanced Recovery after Laser Ablation Surgery Protocol: A Preliminary Analysis. Neuro-Oncol. Pract..

[93] Pruitt R., Gamble A., Black K., Schulder M., Mehta A.D. (2017). Complication Avoidance in Laser Interstitial Thermal Therapy: Lessons Learned. J. Neurosurg..

[94] Smith A.B., Johnson C.D. Efficacy of Drug X in Treating Condition Y: A Randomized Controlled Trial. Clinical Trial. 15 January 2023. https://clinicaltrials.gov/Ct2/Show/NCT05318612.

[95] Gonzalez J.M., Patel R. Evaluation of Drug Z for the Treatment of Disease X: A Phase III Clinical Trial. Clinical Trial. 20 May 2021. https://clinicaltrials.gov/Ct2/Show/NCT02970448.

[96] Chandar J.S., Bhatia S., Ingle S., Mendez Valdez M.J., Maric D., Seetharam D., Desgraves J.F., Govindarajan V., Daggubati L., Merenzon M. (2023). Laser Interstitial Thermal Therapy Induces Robust Local Immune Response for Newly Diagnosed Glioblastoma with Long-Term Survival and Disease Control. J. Immunother..

[97] Campian J., Ghiaseddin A., Rahman M., Huang J., Ansstas G., Kim A., Leuthardt E., Tran D. (2019). ATIM-45. Long Term Follow-Up of a Phase I/II Study Testing the Toxicities and Efficacy of Pembrolizumab in Combination with MRI-Guided Laser Interstitial Thermal Therapy (LITT) in Recurrent Malignant Gliomas. Neuro-Oncology.

[98] Luo M., Chen S.-L., Chen J., Yan H., Qiu Z., Chen G., Lu L., Zhang F. (2020). Resection vs. Ablation for Lesions Characterized as Resectable-Ablative within the Colorectal Liver Oligometastases Criteria: A Propensity Score Matching from Retrospective Study. PeerJ.

[99] Sloan A.E., Ahluwalia M.S., Valerio-Pascua J., Manjila S., Torchia M.G., Jones S.E., Sunshine J.L., Phillips M., Griswold M.A., Clampitt M. (2013). Results of the NeuroBlate System First-in-Humans Phase I Clinical Trial for Recurrent Glioblastoma: Clinical Article. J. Neurosurg..

[100] Wilson H., Chen C. (2023). INNV-29. The Clearpoint Prism^®^ Laser Ablation System: A New Platform for Laser Interstitial Thermal Therapy (LITT) in Neuro-Oncology. Neuro-Oncology.

