# Supplementary material for: Safety and Efficacy of Laser Interstitial Thermal Therapy as Upfront Therapy in Primary Glioblastoma and IDH-Mutant Astrocytoma: A Meta-Analysis
Source: Cancers (Basel). 2024 Jun 3;16(11):2131. doi: 10.3390/cancers16112131 (PMC11171930; doi:10.3390/cancers16112131)
Supplement: Supplementary file 1 [file cancers-16-02131-s001.zip › cancers-3016805-supplementary.pdf]

Supplemental Tables:

Supplemental Table S1. Tumor Location.

Tumor Location	GBM (%)	Astrocytoma (%)
Basal Ganglia	2	0
Butterfly	1	0
Corpus Callosum	15	6
Frontoparietal	19	44
Insular	5	11
Lobar	10	0
Occipital	1	6
Parietooccipital	1	0
Splenium	2	0
Subcortical	4	0
Temporal	8	17
Thalamus	33	17

The tumor occurrence at each location stratified by tumor type. *Note: Daggubati et. al., deGroot et. al., Kamath et. al., and Merenzon et. al. did not report tumor locations and were not included in the IDH-wildtype GBM group. Borghei-Razavi did not report tumor locations and was not included in the IDH-mutant astrocytoma group.*

Supplemental Table S2. Adjuvant Therapy in Combination with LITT.

Adjuvant Therapy	GBM (%)	Astrocytoma (%)
Temozolomide + Radiotherapy	47	-
Dexamethasone (Steroid)**	8	-
Chemotherapy* + Radiotherapy	7	9
Radiotherapy	5	4
Bevacizumab + Radiotherapy	2	-
Lomustine + Radiotherapy	1	-
Tumor Treating Fields	1	-
Temozolomide + Bevacizumab + RT	1	4
Lomustine	1	-
Chemotherapy*	1	4
Pembrolizumab (Immunotherapy)	1	-
Temozolomide + Bevacizumab	1	-
None	51	83

The occurrence of adjuvant therapy by tumor type. *RT: Radiotherapy; *: Specific chemotherapy was not identified by study **: Patients using steroids coincide with patients using other adjuvant therapies.*

Supplemental Figures:

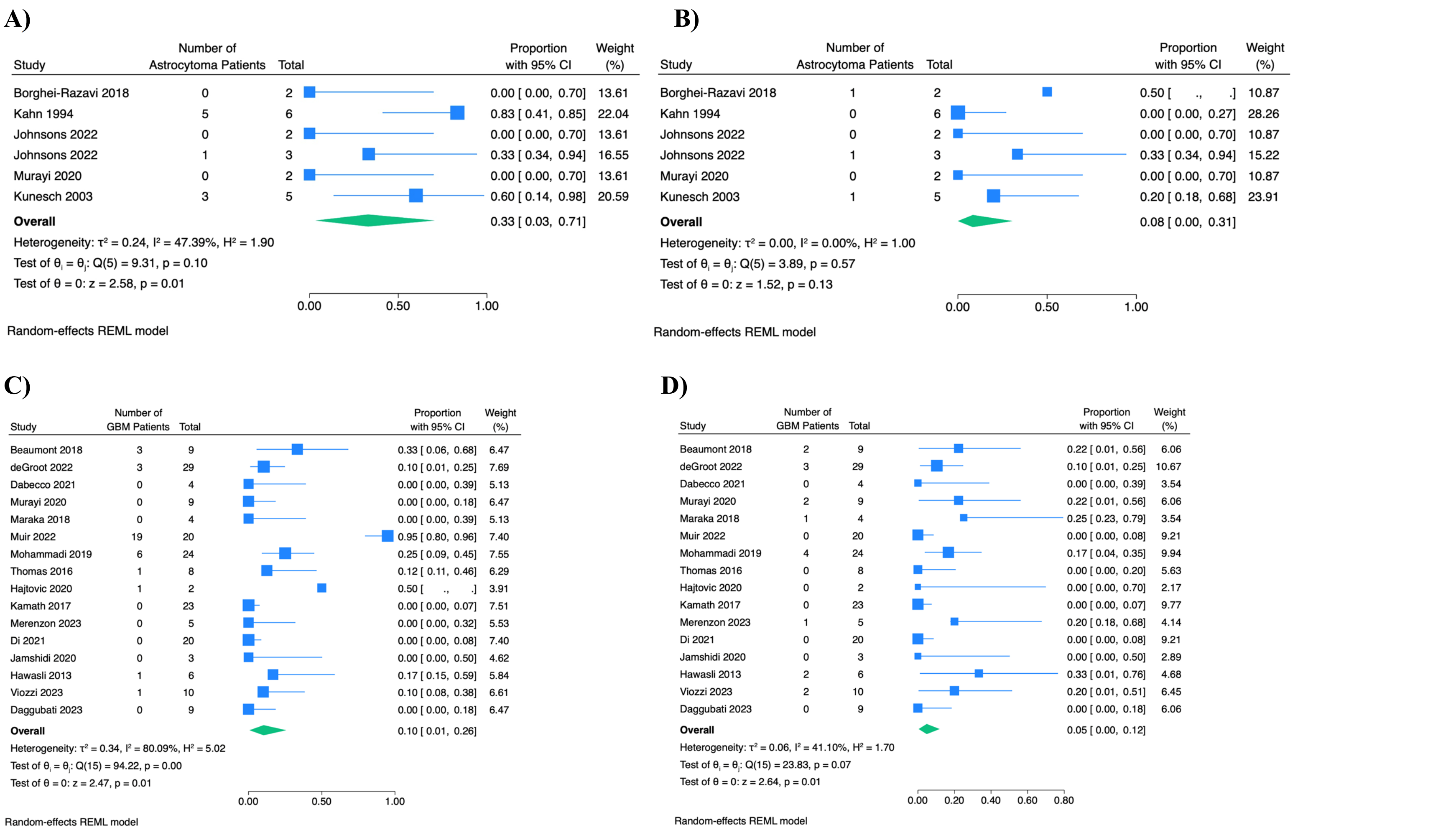

Supplemental Figure S1. Forest Plots for Type of Complication and Tumor. The forest plots with their respective pooled averages for (A) neurologic complications and (B) non-neurologic complications in the IDH-mutant astrocytoma subgroup while the (C) neurologic complications and (D) non-neurologic complications are for the IDH-wildtype GBM subgroup.

A)

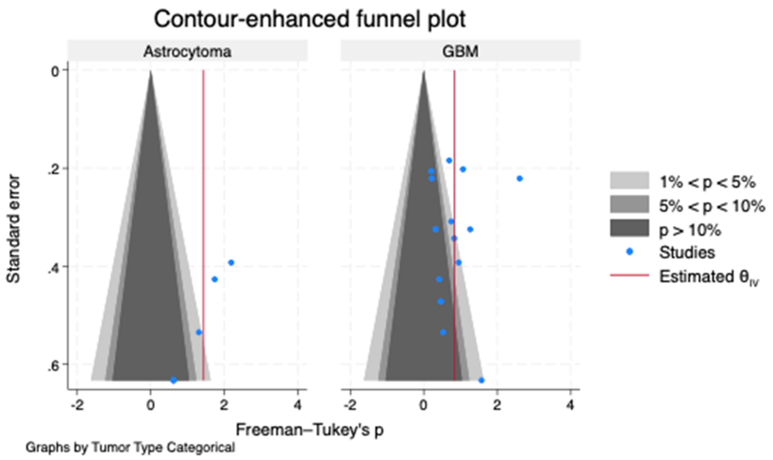

B)

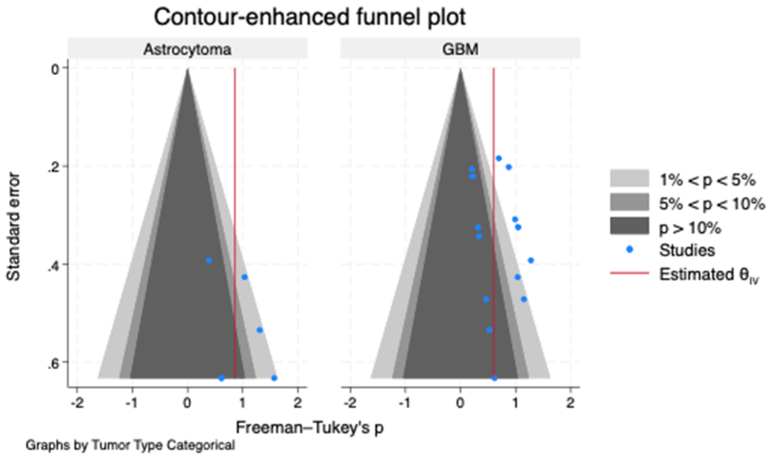

Supplemental Figure S2. Egger's Test for Publication Bias. Egger's test evaluates publication bias and portrays it as a contour-enhanced funnel plot. In this meta-analysis, only the neurologic complications in the IDH-mutant astrocytoma subgroup appeared to have a significant publication bias (p-value < 0.01).
